# Quantity or quality? Assessing relationships between perceived social connectedness and recorded encounters

**DOI:** 10.1371/journal.pone.0208083

**Published:** 2018-11-29

**Authors:** Alison Dias, Nicholas Geard, Patricia Therese Campbell, Deborah Warr, Jodie McVernon

**Affiliations:** 1 Centre for Epidemiology and Biostatistics, Melbourne School of Population and Global Health, The University of Melbourne, Melbourne, Australia; 2 School of Computing and Information Systems, The University of Melbourne, Melbourne, Australia; 3 Victorian Infectious Diseases Reference Laboratory Epidemiology Unit at The Peter Doherty Institute for Infection and Immunity, The University of Melbourne and Royal Melbourne Hospital, Melbourne, Australia; 4 Infection and Immunity Theme, Murdoch Children’s Research Institute, Royal Children’s Hospital, Melbourne, Australia; 5 Centre for Health Equity, Melbourne School of Population and Global Health, The University of Melbourne, Melbourne, Australia; Northwestern University, UNITED STATES

## Abstract

**Introduction:**

Higher levels of social connectedness are associated with better physical and mental health outcomes, but measures of connectedness are often study specific. Prior research has distinguished between perceived and received (quantifiable) measures of social connectedness, with differing impacts on health, sometimes mediated by place of residence. This analysis investigated the relationship between perceptions of social support/connection and quantifiable measures of social encounters, by neighbourhood, to inform understanding of place-based differences in connectedness and health outcomes.

**Methods:**

Negative binomial regression models were used to determine associations between perceptions of social connectedness (perceived community connections and social involvement) and the number of recorded daily social encounters as a proxy for received support/connectedness. Analyses were undertaken across two Local Government Areas (LGAs) in Melbourne with disparate socio-economic profiles to examine potential modification of social connectedness measures by neighbourhood of residence.

**Results:**

Two measures of perceived connectedness had a clear relationship with recorded daily social encounters–feeling a sense of community belonging (RR 1.20 (1.04, 1.37), p = 0.010) and having family or friends close by (RR 1.30 (1.10,1.54), p = 0.002 “neither” compared to “disagree”, (RR 1.15 (1.04, 1.26), p = 0.006 “agree” compared to “disagree”). Involvement in a local church, sporting or social club was associated with a greater number of daily social encounters for respondents who participated a few times a year (RR 1.17 (1.05,1.32), p = 0.006) or often (RR 1.23 (1.12,1.36), p<0.001) compared to never. In the less affluent LGA, active contributions to neighbours and community through assistance and volunteering were a frequent driver of social connection. Differences in patterns between the two areas were found with some measures of perception showing stronger relationships with recorded daily encounters in one area but not the other.

**Conclusions:**

These results indicate substantial complexity in the relationship between perceptions of social connectedness and recorded daily social encounters/received connectedness, meaning that one cannot be reliably extrapolated from the other. Drivers of individuals’ social connections also varied by area of residence. These findings offer new insights into potential mediators of the association between connectedness and wellbeing.

## Introduction

Being socially connected—via both informal relationships, with family and friends, and formal relationships established through workplaces and involvement in faith-based and volunteer organisations—has long been associated with improved health outcomes [1–11]. These benefits include reductions in cancer, cardiovascular and infectious diseases, improved mental health [4, 5] and longer life expectancy [1, 12]. The landmark 1979 Alameda County study found that less connected individuals were more likely to die in the follow-up period than those with more social and community ties [4]. A systematic review undertaken in 2010 reported that individuals with stronger relationships had a 50% increased likelihood of survival [12]. This association is stronger than that between mortality and risk factors such as obesity and physical activity [12].

In their 2015 meta-analysis, Shor and Roelfs summarised three explanations for the positive association between social connectedness and health: a) the moderating effects relationships have in providing emotional support in times of stress and loneliness; b) the potential to facilitate healthy behaviours, and c) the greater availability of emotional and material assistance provided by increased social connectedness [2]. However, it has also been recognised that social relationships are not always positive and can involve demands on individuals that contribute to experiences of stress, conflict or disappointment [5]. There is variation in the type, frequency and intensity of social connections and relationships, and support that is available can differ across relationships [9].

An absence of consistency in relationships between connectedness and health has been addressed by trying to tease out differences between perceived and received social support, measures that are not necessarily correlated [5, 8]. Received support refers to supportive behaviours actually received by an individual, while perceived support incorporates accessibility of and satisfaction with potential sources of support [13]. Perceived support is more closely linked to health outcomes than received support or social network size [5–7], which is a key consideration in researching relationships between social connectedness and health.

Studies of social networks in settings of place-based disadvantage have identified structural differences in the perceived resources available through social connections compared with more affluent areas, indicating that socio-demographic variables and area of residence may be important modifiers of the benefits of social connection [14, 15]. Such effects may explain ‘dose-dependent’ improvements in some health outcomes resulting from connections outside disadvantaged neighbourhoods, potentially by enabling access to a broader pool of information and supports [16].

In this study, we applied standardised measures of social encounters developed by the infectious diseases community as a proxy of received encounters in two local government areas of greater Melbourne with very different socio-demographic profiles. Counts of encounters were compared with questions on perceived connectedness derived from population health and neighbourhood evaluation surveys used in Victoria. We hypothesised that connections would differ by neighbourhood, and also that the relationship between received and perceived connections would be modified by place-based disadvantage.

## Methods

### Study population

Participants were recruited in two geographically distinct local government areas (LGAs) in metropolitan Melbourne, Boroondara and Hume, with contrasting demographic and socioeconomic characteristics. Boroondara is located 5km east of Melbourne’s central business district (CBD), and when the data were collected in 2013, had a median household income for families with dependent children which was greater than AUD$2,500 a week. Residents aged ≥18 years accounted for 79% of the population at the time. Just over a third (34%) of residents were born overseas, with 5% from China and 3% from the United Kingdom. Hume, located at a distance of 20km from the CBD had a median household income for families with dependent children of AUD$1,300 per week at the time of study recruitment. Hume’s population is younger on average than that of Boroondara, with 69% of its residents aged 18 or over. A greater proportion of the population was born overseas (43%), with the most common countries of origin Turkey (5%) and Iraq (5%).

### Data collection

A market research company identified potential participants by random digit dialling within local telephone exchanges. Those who provided verbal consent undertook a computer assisted telephone interview (CATI) lasting 20–30 minutes, during which participants described demographic characteristics of their household and retrospectively provided individually listed information on all social encounters from the previous day, defined as a two-way face-to-face conversation of more than three words, or any physical contact. Only records of individual encounters were included in the analysis. The present study relates those data to a concurrently administered questionnaire on perceptions of connectedness and neighbourhood attributes. Survey instruments are provided in S1 File. Respondents were asked to rate aspects of their community connections and their neighbourhood on 3- or 5-point Likert scales.

### Data analysis

#### Recorded social encounters

Recorded social encounters describe each participant’s reported number of uniquely identified individuals with whom a face-to-face conversational exchange or any physical contact occurred over a 24-hour period. Contacted individuals encountered more than once in the 24-hour period were only recorded once.

#### Perceived community connection

The original responses to the perceived community connections questions were recorded on a scale of Disagree strongly / Disagree / Neither / Agree / Agree strongly / Don’t know. The questions focused on the extent to which participants had connections nearby, felt a sense of belonging to their community and could raise money in an emergency. Responses of ‘don’t know’ were excluded from the analysis. This scale was amalgamated to a 3-point scale of disagree (Disagree strongly and Disagree combined), neither and agree (Agree and Agree Strongly combined) to increase the number of respondents in the categories and align with 3-point scales in the social involvement and perceptions of neighbourhood question sets.

#### Perceived social involvement

For perceived social community involvement, respondents were asked on a scale of Never / A few times / Often / Don’t know, how often they participated in activities over the past year. Responses of ‘don’t know’ were excluded from the analysis.

#### Perceptions of neighbourhood

For the questions on neighbourhood perceptions, participants were asked to rate their neighbourhood and their local community services on a scale of Poor / Average / Good / Don’t know. Responses of ‘don’t know’ were excluded from the analysis.

#### Statistical analysis

Univariate and multivariate negative binomial regression models were used to analyse the relationship between perceived connections / social involvement and recorded social encounters. This approach was deemed most appropriate, given the overdispersion of the recorded social encounters variable in the dataset. All analyses were performed using STATA Version 14.2 (StataCorp, 2015. *Stata Statistical Software*: *Release 14*. College Station, TX: StataCorp LP). Results were reported as rate ratios and a two-tailed P<0.05 was considered statistically significant.

Separate multivariate negative binomial regression models tested the relationship between (i) perceived connections and recorded social encounters, and (ii) perceived involvement and recorded social encounters. These models controlled for gender, age, household size, household income level and educational attainment, as socio-demographic characteristics are independently related to the number of social encounters.

Subgroup analyses were undertaken by local government area to identify any placed-based differences between the responses from residents of Boroondara and Hume.

The potential for participants’ neighbourhood (place-based) perceptions to mediate the relationship between their perceived social connectedness (connections and involvement) and recorded social encounters was also considered. A multivariate negative binomial regression was performed to test the relationship between the neighbourhood perception subset of questions and the number of recorded social encounters. If participants’ perceptions of neighbourhood were shown to be associated with recorded social encounters, they would be considered as a potential confounder in the analysis of perceived community connections/social involvement and recorded social encounters.

### Ethical permissions

The study protocol was approved by the University of Melbourne Human Research Ethics Committee. Participants gave verbal informed consent prior to interview commencement. As the study was conducted via telephone, it was not feasible to obtain written consent from participants. Prospective participants were provided with information about the extent and purpose of the study, and the opportunity to discuss further with a member of the study team. Those interested in proceeding provided verbal consent prior to completion of the interview. This approach was approved by the University of Melbourne Human Research Ethics Committee (Ethics ID 1238477).

## Results

### Study population

Differences by LGA were evident across a number of population characteristics (Table A in S1 Information). The sample population from Boroondara was much older than that from Hume (average age B: 60.5 years; H: 52.7 years), generally had smaller household sizes (B: 2.4 people on average; H: 3.0 people), and higher levels of educational attainment (B: 60% educated to university level; H: 23%). Categorical income data on the sample population showed higher levels of income in Boroondara than Hume (B: 49% earned $1,600 per week or more; H: 29%).

Demographic characteristics of the sample population differed from those of the areas surveyed across a range of factors in both Boroondara and Hume. Compared with 2011 Australian census data, there was an over-representation of individuals who were aged over 50 years, female, Australian-born, English-speaking, and married. Rolls *et al*. provide further detail in their study using the same data [17].

### Recorded social encounters

The number of recorded daily social encounters per person ranged from 1 to 26, with a mean value of 5.6 daily encounters and a standard deviation of 3.9.

### Perceived social connectedness by neighbourhood

A higher proportion of participants from Boroondara responded positively to four of the six perceived community connections measures (Table B in S1 Information). The gap was most evident in whether participants agreed that they could raise $2,000 within 2 days from their relatives and friends; 87% of Boroondara participants agreed, compared to 70% of Hume participants. 80% of those from Boroondara agreed that they know quite a few people who live in their neighbourhood, compared to 75% from Hume, however, 63% of Hume participants agreed that many of their family and friends live in this neighbourhood or close by, a higher proportion than in Boroondara (59%).

Overall, residents from Boroondara had higher levels of social involvement than those from Hume (Table C in S1 Information). A higher proportion of Hume participants indicated that in the past year, they never volunteered with a community organisation than those from Boroondara (59% and 49% respectively). Residents from Boroondara were more likely than Hume residents to have often taken part in a local church, sporting or social club (42% compared to 32%, respectively) and to have often been out to a local café, pub or show (69% compared to 45%, respectively). However, similar proportions were found between Boroondara and Hume in terms of visiting friends locally, and speaking to neighbours.

Participants from Boroondara consistently rated their neighbourhood more positively than those from Hume (Table D in S1 Information). The overwhelming majority of Boroondara residents (98%) rated their neighbourhood as a good place to live, compared to three-quarters of Hume residents (76%). Local community services were rated as “good” by 87% of Boroondara participants, compared to 55% of those from Hume. A similar result was found between the local government areas with regard to access to recreational and leisure facilities, with 89% of Boroondara participants providing a rating of “good”, compared to 57% from Hume.

### Perceived community connections and number of daily social encounters

Of the six community connections questions (Survey instruments provided in S1 File), only two—“I feel a sense of belonging to this community” (RR 1.20 (1.04, 1.37), p = 0.010) and “Many of my friends and family live in this neighbourhood or close by” (“Neither” compared to “Disagree” RR 1.30 (1.10,1.54), p = 0.002, “Agree” compared to “Disagree” (RR 1.15 (1.04, 1.26), p = 0.006)—were clearly related to the number of recorded encounters. Mixed results were evident in both the univariate regression and the multivariate regression in which demographic characteristics were controlled (Table 1).

**Table 1:** Multivariate negative binomial regression results–perceived community connections.

	Overall			Boroondara			Hume		
	RR	95% CI	P-value	RR	95% CI	P-value	RR	95% CI	P-value
**I know quite a few people who live in this neighbourhood**									
Disagree (Reference)									
Neither	0.92	0.74–1.13	0.405	0.94	0.70–1.27	0.699	0.94	0.69–1.28	0.692
Agree	1.02	0.91–1.15	0.718	1.14	0.93–1.40	0.218	0.95	0.82–1.11	0.550
**I feel a sense of belonging to this community**									
Disagree (Reference)									
Neither	1.09	0.91–1.30	0.366	1.25	0.93–1.67	0.134	1.00	0.80–1.26	0.969
Agree	**1.20**	**1.04–1.37**	**0.010**	**1.46**	**1.15–1.86**	**0.002**	1.08	0.91–1.28	0.379
**Many of my friends and family live in this neighbourhood or close by**									
Disagree (Reference)									
Neither	**1.30**	**1.10–1.54**	**0.002**	**1.36**	**1.08–1.71**	**0.010**	1.26	0.98–1.62	0.077
Agree	**1.15**	**1.04–1.26**	**0.006**	**1.23**	**1.07–1.41**	**0.003**	1.07	0.94–1.23	0.298
**I feel generally valued by the community**									
Disagree (Reference)									
Neither	1.04	0.91–1.19	0.048	1.13	0.92–1.39	0.245	0.99	0.82–1.19	0.879
Agree	1.05	0.93–1.18	0.078	1.21	1.00–1.47	0.051	0.96	0.82–1.12	0.620
**I feel I have some influence or control over decisions made in this neighbourhood**									
Disagree (Reference)									
Neither	0.99	0.89–1.11	0.909	1.01	0.86–1.18)	0.894	0.99	0.83–1.17	0.866
Agree	0.94	0.85–1.04	0.208	1.00	0.86–1.15	0.959	0.88	0.77–1.02	0.086
**In an emergency, I could raise $2,000 within 2 days from my relatives and friends**									
Disagree (Reference)									
Neither	0.81	0.63–1.05	0.112	0.95	0.60–1.50	0.823	0.77	0.56–1.04	0.087
Agree	0.97	0.86–1.10	0.671	1.14	0.91–1.44	0.258	0.90	0.77–1.04	0.155

Subgroup analysis by LGA highlighted some differences between Boroondara and Hume. A relationship between agreeing with the statement “I feel a sense of belonging to this community” and a higher number of recorded social encounters was observed for residents of Boroondara (RR 1.46 (1.15,1.86), p = 0.002) but not Hume. Boroondara residents who responded “Neither” (RR 1.36 (1.08,1.71), p = 0.010) or “Agree” (RR 1.23 (1.07,1.41), p = 0.003) to “Many of my friends and family live in this neighbourhood or close by” had more social encounters than those who responded “Disagree”, but this difference was not observed in Hume.

### Perceived social involvement and number of daily social encounters

Analysis of the seven social involvement questions (Survey instruments provided in S1 File) indicates a correlation between greater involvement in social activities and a higher number of social encounters (Table 2). For four of the seven questions, a dose response was evident between frequency of participation in social activities and the number of social encounters. For the question “Over the past year how often have you taken part in a local church, sporting or social club?”, results showed a greater number of daily social encounters for respondents who answered “Yes, a few times” (RR 1.17 (1.05,1.32), p = 0.006) or “Yes, often” (RR 1.23 (1.12,1.36), p<0.001) compared to “Never”. A similar trend was found in the relationship between the question “Over the past year, how often have you been to a public meeting or signed a petition?” and the number of daily social encounters. A greater number of social encounters was associated with responses of “Yes, a few times” (RR 1.11 (1.01,1.21), p = 0.032) and “Yes, often” (RR 1.27 (1.07,1.51), p = 0.006) compared to “Never”.

**Table 2:** Multivariate negative binomial regression results–perceived social involvement.

	Overall			Boroondara			Hume		
	RR	95% CI	P-value	RR	95% CI	P-value	RR	95% CI	P-value
**Over the past year, how often have you done voluntary work with a community organisation?**									
Never (Reference)									
Yes, a few times	**1.15**	**1.04–1.28**	**0.008**	1.08	0.93–1.26	0.328	**1.23**	**1.06–1.42**	**0.006**
Yes, often	**1.15**	**1.04–1.28**	**0.009**	1.11	0.96–1.29	0.172	**1.19**	**1.02–1.39**	**0.028**
**Over the past year, how often have you visited friends locally?**									
Never (Reference)									
Yes, a few times	1.16	0.98–1.38	0.083	1.14	0.89–1.45	0.293	1.25	0.97–1.60	0.085
Yes, often	**1.28**	**1.09–1.51**	**0.003**	1.18	0.94–1.48	0.145	**1.47**	**1.16–1.87**	**0.001**
**Over the past year, how often have you spoken to your neighbours?**									
Never (Reference)									
Yes, a few times	0.95	0.71–1.26	0.708	0.90	0.51–1.58	0.718	0.98	0.70–1.38	0.921
Yes, often	1.15	0.87–1.53	0.334	1.17	0.67–2.05	0.575	1.14	0.82–1.59	0.424
**Over the past year, how often have you minded a friend’s or neighbour’s child?**									
Never (Reference)									
Yes, a few times	**1.22**	**1.08–1.37**	**0.001**	1.09	0.91–1.31	0.342	**1.35**	**1.15–1.58**	**<0.001**
Yes, often	**1.17**	**1.03–1.34**	**0.018**	1.00	0.81–1.23	0.994	**1.29**	**1.09–1.53**	**0.003**
**Over the past year, how often have you taken part in a local church, sporting or social club?**									
Never (Reference)									
Yes, a few times	**1.17**	**1.05–1.32**	**0.006**	**1.21**	**1.02–1.44**	**0.035**	1.15	0.98–1.34	0.085
Yes, often	**1.23**	**1.12–1.36**	**<0.001**	**1.28**	**1.11–1.47**	**0.001**	**1.20**	**1.04–1.38**	**0.011**
**Over the past year, how often have you been out to a local café, pub or show?**									
Never (Reference)									
Yes, a few times	1.10	0.94–1.28	0.237	**1.58**	**1.13–2.21**	**0.008**	0.99	0.83–1.18	0.930
Yes, often	**1.31**	**1.13–1.52**	**<0.001**	**1.81**	**1.31–2.51**	**<0.001**	**1.23**	**1.03–1.46**	**0.019**
**Over the past year, how often have you been to a public meeting or signed a petition?**									
Never (Reference)									
Yes, a few times	**1.11**	**1.01–1.21**	**0.032**	0.99	0.87–1.13	0.889	**1.22**	**1.07–1.40**	**0.003**
Yes, often	**1.27**	**1.07–1.51**	**0.006**	1.25	0.98–1.61	0.076	**1.28**	**1.00–1.62**	**0.046**

General trends across both local government areas were similar for the multivariate analysis of social involvement as a predictor of number of daily social encounters. However, the magnitude of the effect differed between local government areas in some cases (Table 2). For example, the absolute number of contacts increased more for Hume residents who reported visiting local friends than those in Boroondara. The converse relationship is true for the question “Over the past year, I have been out to a local café, pub or show”, an activity more strongly associated with social connections in Boroondara than Hume. In addition, Hume residents were more likely to be socially connected through making proactive contributions to friends and the community, such as volunteering, child-minding or becoming engaged in local issues.

### Neighbourhood perceptions and number of daily social encounters

As evident in Table 3, clear trends were not apparent in the multivariate analysis of neighbourhood perception as a predictor of daily social encounters. Only a rating of “good” regarding perception of the neighbourhood as a place to live was positively associated with an increased number of social encounters. Sub-group analysis by local government area revealed no substantive differences (Table E in S1 Information), although residents of Boroondara who rated services for children and families as “good” recorded somewhat higher levels of connectedness (RR 1.21 (1.05, 1.40), p = 0.008).

**Table 3:** Multivariate negative binomial regression results–neighbourhood perceptions.

	RR	95% CI	P-value
**How would you rate your neighbourhood as a place to live?**			
Poor (Reference)			
Average	1.73	0.99–3.04	0.055
Good	**1.94**	**1.12–3.36**	**0.019**
**How would you rate your local community services?**			
Poor (Reference)			
Average	1.01	0.79–1.28	0.961
Good	1.07	0.85–1.36	0.548
**How would you rate services for families and young children?**			
Poor (Reference)			
Average	1.13	0.90–1.42	0.293
Good	1.08	0.87–1.34	0.502
**How would you rate local health and welfare services?**			
Poor (Reference)			
Average	1.14	0.92–1.42	0.232
Good	1.11	0.91–1.37	0.297
**How would you rate access to recreational and leisure facilities?**			
Poor (Reference)			
Average	0.95	0.79–1.15	0.624
Good	0.97	0.82–1.15	0.747
**How would you rate crime and personal safety in your neighbourhood?**			
Poor (Reference)			
Average	1.12	0.91–139	0.285
Good	1.11	0.90–1.35	0.325

## Discussion

The analyses identified complex relationships between measures of perceived connectedness and belonging, and daily recorded social encounters. Differences found between the two LGAs support other evidence that characteristics of people’s social connections do vary by attributes of the area of residence, including socio-economic profiles. The drivers of quantifiable connection in Hume were activities that involve contributing to others, such as volunteering and minding friends’ or neighbours’ children. In Boroondara, involvement in social events and proximity to friends and family were more strongly associated with the number of social encounters.

Previous work has explored the relationship between perceived and received social connection. In looking at perceived isolation and social disconnectedness, Cornwall and Waite [1] found them to be independently associated with lower levels of self-reported physical health, and as such are not interchangeable indicators. Our study reinforces the distinctness of these alternative indicators of connection. We found no uniform association between perceptions of social connectedness and quantifiable social encounters. This lack of clear association reinforces previous observations that perceived and received connectedness may be independent, and suggests that both quality and quantity of connections may play a role in making people feel supported. Prior work has repeatedly noted the low correlation between perceived and received connectedness [5, 8], which highlights the need to further examine other points of difference, including neighbourhood of residence and the drivers of connection across different groups.

Our results indicate that the behaviours, activities and facilities that foster connection differ between Boroondara and Hume. These differences have implications for urban planning and policy development as they suggest different priorities may need to be addressed in order to create neighbourhoods that support social connectedness. This could stem from differences in tenure in the neighbourhood, socioeconomic position or the built environment, among other factors. There was a much more volitional aspect to the drivers of connection in Hume compared to Boroondara, which may reflect the greater effort required to develop community connection in Hume. Interestingly, while residents of Hume perceived their area as having poorer quality services than Boroondara (and a less favourable place to live), this measure was not found to be associated with quantifiable social encounters. Prior research found an association between low socioeconomic status and limited social networks [18]. This was not observed in our study, which found little difference in overall number of recorded contacts between Boroondara and Hume. Shor and Roelfs’ meta-analysis of social contact and mortality noted that the perceptions and experiences of social relationships are not fully illustrated by examining only objective measures of social connectedness [2]. The analysis of both perceived and objective measures of social connectedness was able to provide further insight into the structure and quality of social connections, which clearly differ across Boroondara and Hume.

A key strength of the study was the individual assessment of the relationships between separate aspects of perceived social connectedness and quantifiable connection (recorded daily social encounters). The mixed results suggest that the differences between these aspects are meaningful, and highlight the need to continue building on previous work around the perceived connectedness-received connectedness dichotomy. Another strength of the study was the analysis from a neighbourhood perspective, facilitated by data from two distinct LGAs. Differences in the drivers of connection between the two areas highlight heterogeneity based on cultural, environmental and spatial diversity.

The study had some limitations. The social encounter measure only accounted for face-to-face encounters, which excluded contact over the telephone, and through social media. Although the social encounters measure was used as a proxy for received social connectedness, it was not possible to distinguish between positive or negative social encounters. The unknown qualitative nature of the encounter limits the interpretation of the results. Computer assisted telephone interviews through local telephone exchanges were used to ensure participants’ residency within one of the two local government areas, however, this approach had limitations in recruitment. It excluded those who did not have landlines, and was limited to participants who were home during the call, which may account for the older sample population. There was also a potential bias in the retrospective collection of social encounter data, in that participant recall may have affected responses, particularly if a participant encountered many contacts that day [19]. Furthermore, participants who declined to participate due to limited English or communication difficulties may have affected the representativeness of the sample and influenced the results, as limited English could be related to lower levels of social connectedness in an English-speaking country.

This study took a broad perspective in looking at daily recorded social encounters, which was a coarse measurement. Further research is needed in this area. Indeed, a 2015 meta-analysis of social contact frequency and all-cause mortality reported that mere social contact frequency (defined as quantity of interactions, without assessing the quality of the support) may not benefit health as much as was previously thought [2], indicating that we need to delve deeper into social connectedness to identify the specific factors that do benefit health. More nuanced work is needed to further illuminate the mechanisms through which social connectedness affects health, as this study has shown that the relationship between qualitative and quantifiable connection is not straightforward.

## Supporting information

S1 File Study questionnaire. Selected data was used in the present study.
(DOC)

S1 Information Table A. Demographics of participants by neighbourhood (Local Government Area).
Table B. Survey frequency results by neighbourhood (Local Government Area)–perceived community connections.
Table C. Survey frequency results by neighbourhood (Local Government Area)–perceived social involvement.
Table D. Survey frequency results by neighbourhood (Local Government Area)–neighbourhood perceptions.
Table E. Multivariate negative binomial regression subgroup analysis results–neighbourhood perceptions.
(DOCX)

S1 Data and dictionary (XLS)
