# Supplementary material for: Quantity or quality? Assessing relationships between perceived social connectedness and recorded encounters
Source: PLoS One. 2018 Nov 29;13(11):e0208083. doi: 10.1371/journal.pone.0208083 (PMC6264807; doi:10.1371/journal.pone.0208083)
Supplement: S1 File — Selected data was used in the present study. (DOC) [file pone.0208083.s001.doc]

**SOCIAL CONNECTEDNESS STUDY - QUESTIONNAIRE**

**Your household**

H1 Please list in the table all the people who usually live in your household:

|  | **First name + initial** | **Gender**  **(circle one)** | **Age (years)** |
| --- | --- | --- | --- |
| **Person 1 (you)** |  | Female/Male |  |
| **Person 2** |  | Female/Male |  |
| **Person 3** |  | Female/Male |  |
| **Person 4** |  | Female/Male |  |
| **Person 5** |  | Female/Male |  |
| **Person 6** |  | Female/Male |  |
| **Person 7** |  | Female/Male |  |
| **Person 8** |  | Female/Male |  |

H2 What is your current marital status (please circle one)?

| Never married | Married | Single | Divorced |
| --- | --- | --- | --- |
| Separated | Widowed | Living with partner | |

H3 What country were you born in?

H4 How long have you been in Australia? years

H5 What is the highest level of education you have completed, either in Australia or overseas? If overseas, please specify which country you were educated in.

Top of Form

|  | **Australia** | **Overseas (specify)** |
| --- | --- | --- |
| Primary school | <INPUT TYPE=\ | <INPUT TYPE=\ |
| Up to year 10 | <INPUT TYPE=\ | <INPUT TYPE=\ |
| Year 11 | <INPUT TYPE=\ | <INPUT TYPE=\ |
| Year 12 | <INPUT TYPE=\ | <INPUT TYPE=\ |
| TAFE Diploma or business college | <INPUT TYPE=\ | <INPUT TYPE=\ |
| University | <INPUT TYPE=\ | <INPUT TYPE=\ |
| Other (please specify) | <INPUT TYPE=\ | <INPUT TYPE=\ |

Bottom of Form

***If you are living with a partner:***

H3A What country was your partner born in?

H4A How long has your partner been in Australia? years

H5A What is the highest level of education your partner has completed, either in Australia or overseas? If overseas, please specify which country they were educated in.

Top of Form

|  | **Australia** | **Overseas (specify)** |
| --- | --- | --- |
| Primary school | <INPUT TYPE=\ | <INPUT TYPE=\ |
| Up to year 10 | <INPUT TYPE=\ | <INPUT TYPE=\ |
| Year 11 | <INPUT TYPE=\ | <INPUT TYPE=\ |
| Year 12 | <INPUT TYPE=\ | <INPUT TYPE=\ |
| TAFE Diploma or business college | <INPUT TYPE=\ | <INPUT TYPE=\ |
| University | <INPUT TYPE=\ | <INPUT TYPE=\ |
| Other (please specify) | <INPUT TYPE=\ | <INPUT TYPE=\ |

Bottom of Form

H6 What languages do you speak at home (please list the main one first)?

1. 2. 3.

H7 Is your home (please circle one):

| Owned outright | Owned with a mortgage | Being purchased under a rent/buy scheme |
| --- | --- | --- |
| Being rented | Being occupied rent free | Public Housing |
| Other (please specify) | | |

H8 Which of these categories contains the combined income of your household (family members living with you) before tax and other deductions are taken out during the last financial year (1 July 2010 to 30 June 2011)?

Top of Form

| **Per Week** | **Per Year** |  |
| --- | --- | --- |
| $2,000 or more | $104,000 or more | <INPUT TYPE=\ |
| $1,600-$1,999 | $83,200-$103,999 | <INPUT TYPE=\ |
| $1,300-$1,599 | $67,600-$83,199 | <INPUT TYPE=\ |
| $1,000-$1,299 | $52,000-$67,599 | <INPUT TYPE=\ |
| $800-$999 | $41,600-$51,999 | <INPUT TYPE=\ |
| $600-$799 | $31,200-$41,599 | <INPUT TYPE=\ |
| $400-$599 | $20,800-$31,199 | <INPUT TYPE=\ |
| $250-$399 | $13,000-$20,799 | <INPUT TYPE=\ |
| $150-$249 | $7,800-$12,999 | <INPUT TYPE=\ |
| $1-$149 | $1-$7,799 | <INPUT TYPE=\ |
| Nil income | Nil income | <INPUT TYPE=\ |

Bottom of Form

**Your neighbourhood**

N1 How long have you lived at this address? years

N2 Why did you come to live here (please circle all that apply)?

| Cost of housing | Friends and family lived here | Employment reasons |
| --- | --- | --- |
| Liked the area | Government allocated the house | No other housing available |
| Other (specify) | | |

N3 Where did you live before you came to this address (please circle one)?

| This general area | Some other area |
| --- | --- |

N4 What do you like about living in your neighbourhood (please circle any that apply)?

| Family, friends, a sense of belonging | Convenient to shops, services, facilities | Access to public transport |
| --- | --- | --- |
| Quiet and pleasant neighbourhood | Quality of local services, facilities | Multicultural neighbourhood |
| Aspects of housing (affordability, type) | Parks and open spaces | Safe and healthy environment |
| Other factors (specify) | | |

N5 Is there anything you dislike about living in your neighbourhood?

*Please circle one response to rate the following aspects of your neighbourhood:*

N6 How would you rate your neighbourhood as a place to live?

| Good | Average | Poor | Don’t know |
| --- | --- | --- | --- |

N7 How would you rate local community services?

| Good | Average | Poor | Don’t know |
| --- | --- | --- | --- |

N8 How would you rate services for families and young children?

| Good | Average | Poor | Don’t know |
| --- | --- | --- | --- |

N9 How would you rate local health and welfare services?

| Good | Average | Poor | Don’t know |
| --- | --- | --- | --- |

N10 How would you rate access to recreational and leisure facilities?

| Good | Average | Poor | Don’t know |
| --- | --- | --- | --- |

N11 How would you rate crime and personal safety in your neighbourhood?

| Good | Average | Poor | Don’t know |
| --- | --- | --- | --- |

*How do you feel about the following crime and safety issues (please circle one response)?*

N12 I feel safe walking alone down my street

| Disagree strongly | Disagree | Neither | Agree | Agree strongly | Don’t know |
| --- | --- | --- | --- | --- | --- |

N13 Children can safely play outside in my neighbourhood

| Disagree strongly | Disagree | Neither | Agree | Agree strongly | Don’t know |
| --- | --- | --- | --- | --- | --- |

N14 I can trust most people in this neighbourhood

| Disagree strongly | Disagree | Neither | Agree | Agree strongly | Don’t know |
| --- | --- | --- | --- | --- | --- |

N15 Neighbours around here generally look out for each other

| Disagree strongly | Disagree | Neither | Agree | Agree strongly | Don’t know |
| --- | --- | --- | --- | --- | --- |

**Your connections**

*How do you feel about the following statements (please circle one response)?*

C1 I know quite a few people who live in this neighbourhood

| Disagree strongly | Disagree | Neither | Agree | Agree strongly | Don’t know |
| --- | --- | --- | --- | --- | --- |

C2 I feel a sense of belonging to this community

| Disagree strongly | Disagree | Neither | Agree | Agree strongly | Don’t know |
| --- | --- | --- | --- | --- | --- |

C3 Many of my family and friends live in this neighbourhood or close by

| Disagree strongly | Disagree | Neither | Agree | Agree strongly | Don’t know |
| --- | --- | --- | --- | --- | --- |

C4 I feel generally valued by the community

| Disagree strongly | Disagree | Neither | Agree | Agree strongly | Don’t know |
| --- | --- | --- | --- | --- | --- |

C5 I feel I have some influence or control over decisions made in this neighbourhood

| Disagree strongly | Disagree | Neither | Agree | Agree strongly | Don’t know |
| --- | --- | --- | --- | --- | --- |

C6 In an emergency, I could raise $2,000 within 2 days from my relatives and friends

| Disagree strongly | Disagree | Neither | Agree | Agree strongly | Don’t know |
| --- | --- | --- | --- | --- | --- |

*Over the past year, how often have you:*

C7 Done voluntary work with a community organization?

| Yes, Often | Yes, a few times | Never | Don’t know |
| --- | --- | --- | --- |

C8 Visited friends locally?

| Yes, Often | Yes, a few times | Never | Don’t know |
| --- | --- | --- | --- |

C9 Spoken to your neighbours?

| Yes, Often | Yes, a few times | Never | Don’t know |
| --- | --- | --- | --- |

C10 Minded a friend’s or neighbour’s child?

| Yes, Often | Yes, a few times | Never | Don’t know |
| --- | --- | --- | --- |

C11 Taken part in a local church, sporting or social club?

| Yes, Often | Yes, a few times | Never | Don’t know |
| --- | --- | --- | --- |

C12 Been out to a local café, pub or show?

| Yes, Often | Yes, a few times | Never | Don’t know |
| --- | --- | --- | --- |

C13 Been to a public meeting or signed a petition?

| Yes, Often | Yes, a few times | Never | Don’t know |
| --- | --- | --- | --- |

**Your electronic connections**

E1 Does your household have:

| Internet access | Cable TV | Satellite TV |
| --- | --- | --- |

E2 Which of the following forms of electronic communication do you use?

| Email | Video calling  eg Skype |
| --- | --- |
| Social networking  eg Facebook | Blogging  eg Twitter |
| Other (specify) | |

E3 If you answered yes to any of the above forms of communication, who do you use them to communicate with?

| Family overseas | Family in Australia |
| --- | --- |
| Friends in Australia | Friends overseas |

**Connections and information**

I1 If you had a concern about your health, where would you seek information?

1.

2.

3.

4.

5.
