# Supplementary material for: Quantity or quality? Assessing relationships between perceived social connectedness and recorded encounters
Source: PLoS One. 2018 Nov 29;13(11):e0208083. doi: 10.1371/journal.pone.0208083 (PMC6264807; doi:10.1371/journal.pone.0208083)
Supplement: S1 Information — Table A. Demographics of participants by neighbourhood (Local Government Area). Table B. Survey frequency results by neighbourhood (Local Government Area)–perceived community connections. Table C. Survey frequency results by neighbourhood (Local Government Area)–perceived social involvement. Table D. Survey frequency results by neighbourhood (Local Government Area)–neighbourhood perceptions. Table E. Multivariate negative binomial regression subgroup analysis results–neighbourhood perceptions. (DOCX) [file pone.0208083.s002.docx]

**Table A.** Demographics of participants by neighbourhood (Local Government Area)

|  | **Boroondara** | **Hume** |
| --- | --- | --- |
| Number of participants n | 650 | 657 |
| Recorded social encounters mean (SD) | 5.92 (4.02) | 5.35 (3.84) |
| Gender n (%) |  |  |
| Male | 252 (38.77) | 262 (39.88) |
| Female | 398 (61.23) | 395 (60.12) |
| Total | 650 (100) | 657 (100) |
| Age group (years) n (%) |  |  |
| 18–29 | 49 (7.79) | 76 (11.78) |
| 30–39 | 20 (3.18) | 68 (10.54) |
| 40–49 | 78 (12.40) | 111 (17.21) |
| 50–59 | 128 (20.35) | 154 (23.88) |
| 60–69 | 155 (24.64) | 129 (20.00) |
| 70+ | 199 (31.64) | 107 (16.59) |
| Total | 629 (100) | 645 (100) |
| Missing | 21 | 12 |
| Household size n (%) |  |  |
| 1 person | 185 (28.46) | 92 (14.00) |
| 2 people | 240 (36.92) | 213 (32.42) |
| 3 people | 74 (11.38) | 114 (17.35) |
| 4 people | 97 (14.92) | 143 (21.77) |
| 5 + people | 54 (8.31) | 95 (14.46) |
| Total | 650 (100) | 657 (100) |
| Household income level n (%) |  |  |
| $1,600 or more per week | 215 (48.75) | 137 (28.96) |
| $1,000–$1,500 per week | 92 (20.86) | 122 (25.79) |
| $600–$999 per week | 64 (14.51) | 102 (21.56) |
| $250–$599 per week | 61 (13.83) | 89 (18.82) |
| $150–$249 per week | 9 (2.04) | 23 (4.86) |
| Total | 441 (100) | 473 (100) |
| Missing | 209 | 184 |
| Educational attainment n (%) |  |  |
| University | 391 (60.25) | 148 (22.73) |
| TAFE Diploma or business college | 78 (12.02) | 145 (22.27) |
| Up to Year 12 | 135 (20.80) | 207 (31.80) |
| Up to Year 10 | 37 (5.70) | 112 (17.20) |
| Primary school | 8 (1.23) | 39 (5.99) |
| Total | 649 (100) | 651 (100) |
| Missing | 1 | 6 |
|  |  |  |

**Table B.** Survey frequency results by neighbourhood (Local Government Area) – perceived community connections

I know quite a few people who live in this neighbourhood

| Response | Boroondara (count) | Hume (count) | Total (count) | Boroondara (%) | Hume (%) | Total (%) |
| --- | --- | --- | --- | --- | --- | --- |
| *Disagree* | 84 | 129 | 213 | 13.00 | 19.72 | 16.38 |
| *Neither* | 46 | 33 | 79 | 7.12 | 5.05 | 6.08 |
| *Agree* | 516 | 492 | 1,008 | 79.88 | 75.23 | 77.54 |
| Total | 646 | 654 | 1,300 | 100 | 100 | 100 |
| *Don’t know/Refused* | 4 | 3 | 7 | - | - | - |

I feel a sense of belonging to this community

| Response | Boroondara (count) | Hume (count) | Total (count) | Boroondara (%) | Hume (%) | Total (%) |
| --- | --- | --- | --- | --- | --- | --- |
| *Disagree* | 61 | 101 | 162 | 9.41 | 15.56 | 12.49 |
| *Neither* | 69 | 77 | 146 | 10.65 | 11.86 | 11.26 |
| *Agree* | 518 | 417 | 989 | 79.94 | 72.57 | 76.25 |
| Total | 648 | 649 | 1,297 | 100 | 100 | 100 |
| *Don’t know/Refused* | 2 | 8 | 10 | - | - | - |

Many of my friends and family live in this neighbourhood or close by

| Response | Boroondara (count) | Hume (count) | Total (count) | Boroondara (%) | Hume (%) | Total (%) |
| --- | --- | --- | --- | --- | --- | --- |
| *Disagree* | 201 | 201 | 402 | 31.26 | 30.78 | 31.02 |
| *Neither* | 63 | 40 | 103 | 9.80 | 6.13 | 7.95 |
| *Agree* | 379 | 412 | 791 | 58.94 | 63.09 | 61.03 |
| Total | 643 | 653 | 1,296 | 100 | 100 | 100 |
| *Don’t know/Refused* | 7 | 4 | 11 | - | - | - |

I feel generally valued by the community

| Response | Boroondara (count) | Hume (count) | Total (count) | Boroondara (%) | Hume (%) | Total (%) |
| --- | --- | --- | --- | --- | --- | --- |
| *Disagree* | 97 | 153 | 250 | 15.90 | 24.32 | 20.18 |
| *Neither* | 180 | 136 | 316 | 29.51 | 21.62 | 25.50 |
| *Agree* | 333 | 340 | 673 | 54.59 | 54.05 | 54.32 |
| Total | 610 | 629 | 1,239 | 100 | 100 | 100 |
| *Don’t know/Refused* | 40 | 28 | 68 | - | - | - |

I feel I have some influence or control over decisions made in this neighbourhood

| Response | Boroondara (count) | Hume (count) | Total (count) | Boroondara (%) | Hume (%) | Total (%) |
| --- | --- | --- | --- | --- | --- | --- |
| *Disagree* | 268 | 377 | 645 | 42.68 | 59.09 | 59.95 |
| *Neither* | 138 | 95 | 233 | 21.97 | 14.89 | 18.40 |
| *Agree* | 222 | 166 | 388 | 35.35 | 26.02 | 30.65 |
| Total | 628 | 638 | 1,266 | 100 | 100 | 100 |
| *Don’t know/Refused* | 22 | 19 | 41 | - | - | - |

In an emergency, I could raise $2,000 within 2 days from my relatives and friends

| Response | Boroondara (count) | Hume (count) | Total (count) | Boroondara (%) | Hume (%) | Total (%) |
| --- | --- | --- | --- | --- | --- | --- |
| *Disagree* | 56 | 151 | 207 | 9.35 | 24.43 | 17.01 |
| *Neither* | 22 | 38 | 60 | 3.67 | 6.15 | 4.93 |
| *Agree* | 521 | 429 | 950 | 86.98 | 69.42 | 78.06 |
| Total | 599 | 618 | 1,217 | 100 | 100 | 100 |
| *Don’t know/Refused* | 51 | 39 | 90 | - | - | - |
|  |  |  |  |  |  |  |

In an emergency, I could raise $2,000 within 2 days from my relatives and friends

| Response | Boroondara (count) | Hume (count) | Total (count) | Boroondara (%) | Hume (%) | Total (%) |
| --- | --- | --- | --- | --- | --- | --- |
| *Disagree* | 56 | 151 | 207 | 9.35 | 24.43 | 17.01 |
| *Neither* | 22 | 38 | 60 | 3.67 | 6.15 | 4.93 |
| *Agree* | 521 | 429 | 950 | 86.98 | 69.42 | 78.06 |
| Total | 599 | 618 | 1,217 | 100 | 100 | 100 |
| *Don’t know/Refused* | 51 | 39 | 90 | - | - | - |
|  |  |  |  |  |  |  |

**Table C.** Survey frequency results by neighbourhood (Local Government Area) – perceived social involvement

Over the past year, how often have you done voluntary work with a community organisation?

| Response | Boroondara (count) | Hume (count) | Total (count) | Boroondara (%) | Hume (%) | Total (%) |
| --- | --- | --- | --- | --- | --- | --- |
| *Never* | 316 | 386 | 702 | 48.62 | 58.84 | 53.75 |
| *A few times* | 163 | 147 | 310 | 25.08 | 22.41 | 23.74 |
| *Often* | 171 | 123 | 294 | 26.31 | 18.75 | 22.51 |
| Total | 650 | 656 | 1,306 | 100 | 100 | 100 |
| *Don’t know/Refused* | 0 | 1 | 1 | - | - | - |

Over the past year, how often have you visited friends locally?

| Response | Boroondara (count) | Hume (count) | Total (count) | Boroondara (%) | Hume (%) | Total (%) |
| --- | --- | --- | --- | --- | --- | --- |
| *Never* | 66 | 63 | 129 | 10.20 | 9.62 | 9.91 |
| *A few times* | 199 | 182 | 381 | 30.76 | 27.79 | 29.26 |
| *Often* | 382 | 410 | 792 | 59.04 | 62.60 | 60.83 |
| Total | 647 | 655 | 1,302 | 100 | 100 | 100 |
| *Don’t know/Refused* | 3 | 2 | 5 | - | - | - |

Over the past year, how often have you spoken to your neighbours?

| Response | Boroondara (count) | Hume (count) | Total (count) | Boroondara (%) | Hume (%) | Total (%) |
| --- | --- | --- | --- | --- | --- | --- |
| *Never* | 9 | 22 | 31 | 1.39 | 3.35 | 2.28 |
| *A few times* | 168 | 192 | 360 | 25.93 | 29.27 | 27.61 |
| *Often* | 471 | 442 | 913 | 72.69 | 67.38 | 70.02 |
| Total | 648 | 656 | 1,304 | 100 | 100 | 100 |
| *Don’t know/Refused* | 2 | 1 | 3 | - | - | - |

Over the past year, how often have you minded a friend’s or neighbour’s child?

| Response | Boroondara (count) | Hume (count) | Total (count) | Boroondara (%) | Hume (%) | Total (%) |
| --- | --- | --- | --- | --- | --- | --- |
| *Never* | 485 | 457 | 942 | 75.08 | 69.77 | 72.41 |
| *A few times* | 90 | 108 | 198 | 13.93 | 16.49 | 15.22 |
| *Often* | 71 | 90 | 161 | 10.99 | 13.74 | 12.38 |
| Total | 646 | 655 | 1,301 | 100 | 100 | 100 |
| *Don’t know/Refused* | 4 | 2 | 6 | - | - | - |

Over the past year how often have you taken part in a local church, sporting or social club?

| Response | Boroondara (count) | Hume (count) | Total (count) | Boroondara (%) | Hume (%) | Total (%) |
| --- | --- | --- | --- | --- | --- | --- |
| *Never* | 264 | 306 | 570 | 40.68 | 46.65 | 43.68 |
| *A few times* | 113 | 137 | 250 | 17.68 | 20.88 | 19.16 |
| *Often* | 272 | 213 | 485 | 41.91 | 32.47 | 37.16 |
| Total | 649 | 656 | 1,305 | 100 | 100 | 100 |
| *Don’t know/Refused* | 1 | 1 | 2 | - | - | - |

Over the past year, how often have you been out to a local café, pub or show?

| Response | Boroondara (count) | Hume (count) | Total (count) | Boroondara (%) | Hume (%) | Total (%) |
| --- | --- | --- | --- | --- | --- | --- |
| *Never* | 36 | 115 | 151 | 5.56 | 17.61 | 11.61 |
| *A few times* | 168 | 246 | 414 | 25.93 | 37.67 | 31.82 |
| *Often* | 444 | 292 | 736 | 68.52 | 44.72 | 56.57 |
| Total | 648 | 653 | 1,301 | 100 | 100 | 100 |
| *Don’t know/Refused* | 2 | 4 | 6 | - | - | - |

Over the past year, how often have you been a to a public meeting or signed a petition?

| Response | Boroondara (count) | Hume (count) | Total (count) | Boroondara (%) | Hume (%) | Total (%) |
| --- | --- | --- | --- | --- | --- | --- |
| *Never* | 349 | 434 | 783 | 53.69 | 66.46 | 60.09 |
| *A few times* | 258 | 183 | 441 | 39.69 | 28.02 | 33.84 |
| *Often* | 43 | 36 | 79 | 6.62 | 5.51 | 6.06 |
| Total | 650 | 653 | 1,303 | 100 | 100 | 100 |
| *Don’t know/Refused* | 0 | 4 | 4 | - | - | - |
|  |  |  |  |  |  |  |

**Table D.** Survey frequency results by neighbourhood (Local Government Area) – neighbourhood perceptions

How would you rate your neighbourhood as a place to live?

| Response | Boroondara (count) | Hume (count) | Total (count) | Boroondara (%) | Hume (%) | Total (%) |
| --- | --- | --- | --- | --- | --- | --- |
| *Poor* | 2 | 10 | 12 | 0.31 | 1.52 | 0.92 |
| *Average* | 13 | 149 | 162 | 2.00 | 22.68 | 12.40 |
| *Good* | 634 | 498 | 1,132 | 97.69 | 75.80 | 86.68 |
| Total | 649 | 657 | 1,306 | 100 | 100 | 100 |
| *Don’t know/Refused* | 1 | 0 | 1 | - | - | - |

How would you rate your local community services?

| Response | Boroondara (count) | Hume (count) | Total (count) | Boroondara (%) | Hume (%) | Total (%) |
| --- | --- | --- | --- | --- | --- | --- |
| *Poor* | 2 | 43 | 45 | 0.33 | 6.90 | 3.67 |
| *Average* | 75 | 234 | 309 | 12.42 | 37.56 | 25.18 |
| *Good* | 527 | 346 | 873 | 87.25 | 55.54 | 71.15 |
| Total | 604 | 623 | 1,227 | 100 | 100 | 100 |
| *Don’t know/Refused* | 46 | 34 | 80 | - | - | - |

How would you rate services for families and young children?

| Response | Boroondara (count) | Hume (count) | Total (count) | Boroondara (%) | Hume (%) | Total (%) |
| --- | --- | --- | --- | --- | --- | --- |
| *Poor* | 0 | 52 | 52 | 0.00 | 9.96 | 5.21 |
| *Average* | 63 | 186 | 249 | 13.21 | 35.63 | 24.92 |
| *Good* | 414 | 284 | 698 | 86.79 | 54.41 | 69.87 |
| Total | 477 | 522 | 999 | 100 | 100 | 100 |
| *Don’t know/Refused* | 173 | 135 | 308 | - | - | - |

How would you rate local health and welfare services?

| Response | Boroondara (count) | Hume (count) | Total (count) | Boroondara (%) | Hume (%) | Total (%) |
| --- | --- | --- | --- | --- | --- | --- |
| *Poor* | 5 | 58 | 63 | 0.90 | 9.31 | 5.34 |
| *Average* | 79 | 183 | 262 | 14.18 | 29.37 | 22.20 |
| *Good* | 473 | 382 | 855 | 84.92 | 61.32 | 72.46 |
| Total | 557 | 623 | 1,180 | 100 | 100 | 100 |
| *Don’t know/Refused* | 93 | 34 | 127 | - | - | - |

How would you rate access to recreational and leisure facilities?

| Response | Boroondara (count) | Hume (count) | Total (count) | Boroondara (%) | Hume (%) | Total (%) |
| --- | --- | --- | --- | --- | --- | --- |
| *Poor* | 8 | 79 | 87 | 1.28 | 12.58 | 6.95 |
| *Average* | 59 | 194 | 253 | 9.47 | 30.89 | 20.22 |
| *Good* | 556 | 355 | 911 | 89.25 | 56.53 | 72.82 |
| Total | 623 | 628 | 1,251 | 100 | 100 | 100 |
| *Don’t know/Refused* | 27 | 29 | 56 | - | - | - |

How would you rate personal safety in your neighbourhood?

| Response | Boroondara (count) | Hume (count) | Total (count) | Boroondara (%) | Hume (%) | Total (%) |
| --- | --- | --- | --- | --- | --- | --- |
| *Poor* | 5 | 60 | 65 | 0.77 | 9.26 | 5.02 |
| *Average* | 97 | 193 | 290 | 14.99 | 29.78 | 22.39 |
| *Good* | 545 | 395 | 940 | 84.23 | 60.96 | 72.59 |
| Total | 647 | 648 | 1,295 | 100 | 100 | 100 |
| *Don’t know/Refused* | 3 | 9 | 12 | - | - | - |

**Table E.** Multivariate negative binomial regression subgroup analysis results – neighbourhood perceptions

|  | **Boroondara** | | | **Hume** | | |
| --- | --- | --- | --- | --- | --- | --- |
|  | **RR** | **95% CI** | **P-value** | **RR** | **95% CI** | **P-value** |
| **How would you rate your neighbourhood as a place to live?** | | | | | | |
| Poor (Reference) | | | | | | |
| Average | 3.04 | 0.79-11.73 | 0.107 | 1.60 | 0.85-3.00 | 0.143 |
| Good | 2.78 | 0.84-9.20 | 0.094 | 1.79 | 0.96-3.33 | 0.068 |
| **How would you rate your local community services?** | | | | | | |
| Poor (Reference) | | | | | | |
| Average | 1.48 | 0.60-3.67 | 0.393 | 0.98 | 0.76-1.26 | 0.860 |
| Good | 1.53 | 0.63-3.72 | 0.347 | 1.06 | 0.83-1.36 | 0.650 |
| **How would you rate services for families and young children?** | | | | | | |
| Poor (Reference) | | | | | | |
| Average | 0.84 | 0.69-1.03 | 0.090 | 1.09 | 0.86-1.38 | 0.477 |
| Good | **1.21** | **1.05-1.40** | **0.008** | 1.10 | 0.88-1.38 | 0.391 |
| **How would you rate local health and welfare services?** | | | | | | |
| Poor (Reference) | | | | | | |
| Average | 2.03 | 0.92-4.46 | 0.079 | 1.08 | 0.86-1.37 | 0.493 |
| Good | 1.82 | 0.84-3.93 | 0.129 | 1.08 | 0.87-1.34 | 0.514 |
| **How would you rate access to recreational and leisure facilities?** | | | | | | |
| Poor (Reference) | | | | | | |
| Average | 0.87 | 0.49-1.53 | 0.621 | 0.93 | 0.77-1.14 | 0.503 |
| Good | 0.79 | 0.46-1.37 | 0.406 | 0.97 | 0.81-1.17 | 0.725 |
| **How would you rate personal safety in your neighbourhood?** | | | | | | |
| Poor (Reference) | | | | | | |
| Average | 1.13 | 0.60-2.14 | 0.702 | 1.10 | 0.87-1.38 | 0.420 |
| Good | 1.02 | 0.55-1.91 | 0.945 | 1.12 | 0.90-1.39 | 0.310 |
